# Sex differences in the association between infant markers and later autistic traits

**DOI:** 10.1186/s13229-016-0081-0

**Published:** 2016-03-30

**Authors:** Rachael Bedford, Emily J. H. Jones, Mark H. Johnson, Andrew Pickles, Tony Charman, Teodora Gliga

**Affiliations:** Biostatistics Department, Institute of Psychiatry, Psychology & Neuroscience, King’s College London, London, UK; Centre for Brain and Cognitive Development, Birkbeck College, University of London, London, UK; Psychology Department, Institute of Psychiatry, Psychology & Neuroscience, King’s College London, London, UK

**Keywords:** Sex difference, Infants, Autism, High risk, Differential liability

## Abstract

**Background:**

Although it is well established that the prevalence of autism spectrum disorder (ASD) is higher in males than females, there is relatively little understanding of the underlying mechanisms and their developmental time course. Sex-specific protective or risk factors have often been invoked to explain these differences, but such factors are yet to be identified.

**Methods:**

We take a developmental approach, using a prospective sample of 104 infants at high and low familial risk for ASD, to characterise sex differences in infant markers known to predict emerging autism symptoms. We examine three markers previously shown to be associated with later autistic social-communication symptoms: the Autism Observation Scale for Infants (AOSI) total score, attention disengagement speed and gaze following behaviour. Our aim was to test whether sex differences were already present in these markers at 1 year of age, which would suggest sex-specific mechanisms of risk or protection.

**Results:**

While no sex differences were found in any of the three markers investigated, we found sex differences in their relationship to 3-year autism traits; all three markers significantly predicted later autism traits *only in the boys*.

**Conclusions:**

Previously identified ‘early autism markers’ were associated with later autism symptoms only in boys. This suggests that there may be additional moderating risk or protective factors which remain to be identified. Our findings have important implications for prospective studies in terms of directly testing for the moderating effect of sex on emerging autistic traits.

**Electronic supplementary material:**

The online version of this article (doi:10.1186/s13229-016-0081-0) contains supplementary material, which is available to authorized users.

## Background

It is well established that autism spectrum disorder (ASD) is more prevalent in males than in females, with 1:42 boys and 1:189 girls meeting ASD criteria [1]. Despite this being a topic of great interest (see recent special issue in Molecular Autism, vol. 6), the mechanisms underlying sex differences in the prevalence of autism, as well as in other childhood-onset psychiatric conditions, are still poorly understood [2]. In order to characterise sex differences in infant markers previously shown to predict emerging symptoms of autism, we take a developmental approach using a prospective sample of infants at high risk for ASD. The aim of the current paper is to assess whether sex differences are apparent in known early autism markers or in the relationships between early markers and later autistic traits.

**Fig. 1:**
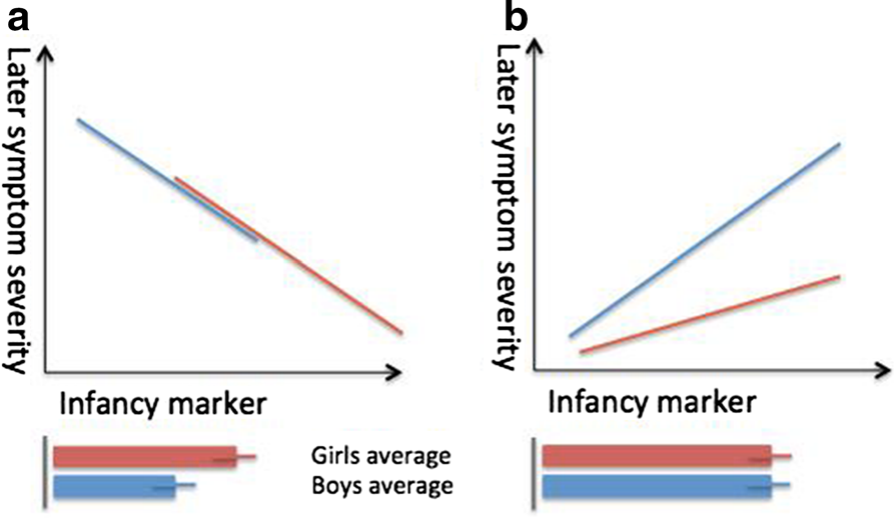
Sex differences can appear in the expression of early autism markers or in the relationship between marker and outcome. **a** The marker differentiates between the boys and girls, but relates to later symptoms in the same way in both sexes; here, having lower levels of this marker is protective in girls. **b** The early marker is similarly expressed in boys and girls but only relates to later symptoms in boys; this is due to additional moderating factors (which are likely to be type of marker described in **a**) decreasing the impact of this marker in girls. A combination of the two models is also possible

According to a prominent hypothesis, the differential liability model [3], boys are more susceptible than girls because they are subject to boy-specific risk factors or because girls benefit from protective factors. To understand the underlying mechanisms, we can examine two types of evidence [2]: direct evidence for sex differences in the risk/protective factors themselves (Fig. 1a), or indirect evidence, in the form of differential relationships between risk/protective factors and the disorder, suggesting the presence of additional sex-specific moderating factors (Fig. 1b). Indirect evidence for girl-specific protective factors comes from studies showing that a higher load of genetic/phenotypic risk is needed for girls to eventually express autistic traits [3]. As a consequence of this, siblings of girls with autism show a higher incidence of autism than siblings of boys with autism [4], although see [5, 6] for no difference in incidence between these groups.

Phenotypic differences between males and females with autism may also reflect the moderating role of sex-specific factors, although evidence for sex differences is mixed. One commonly reported finding is that females with autism have lower IQ than males ([7, 8], although see [9]), which is consistent with the idea of sex differences in the liability threshold. Language has often been suggested as a protective factor, since girls lead in language skills from early in development (e.g. [10]). However, evidence suggests that autistic girls with better language also have better cognitive and adaptive skills, which is not consistent with the idea of protection (e.g. [11, 12]). Evidence from earlier in development is less clear still, with Carter et al. [13] showing significantly better language and fine motor skills in autistic toddler boys but greater visual reception abilities in girls on the Mullen Scales of Early Learning (MSEL; [14]). Other studies find no sex difference in the adaptive and behavioural functioning of toddlers with autism [15, 16]. Differences between studies in the age groups tested, in functional level and in potential diagnostic and sampling biases may cloud the evidence for the differential liability model in autism.

Longitudinal prospective designs of high-risk populations deal with some of these methodological issues, avoiding ascertainment bias and including a typically developing control group. In addition, they allow us to look for sex differences early on in development. Given the evidence for differential genetic liability (e.g. [17]) and the fact that sex differences in prenatal hormones like testosterone have been linked to later autism symptomatology [2], it is plausible that sex-specific risk factors act even before birth, in which case their effects would be apparent in infancy. Messinger et al. [5] and Zwaigenbaum et al. [18] have utilised a prospective longitudinal design with infants at high risk for autism (owing to an older sibling with a diagnosis) to investigate sex differences in the MSEL. Both studies found increased MSEL scores in girls compared to boys across both the high- and low-risk groups. Similarly, Charwarska et al. [19] found that high-risk girls (as compared to both high-risk boys and all low-risk controls) show increased attention to social stimuli. This suggests that autism risk may be differentially expressed in males and females, conferring protection in the girls. As well as allowing the investigation of sex-specific effects very early in development, these studies also have the advantage of a typically developing comparison group, which enables the specificity of sex differences in the development of autism to be directly tested.

Among familial high-risk infants, approximately 20 % go on to an autism diagnosis [20] with a further 20 % showing sub-threshold symptoms [21] and a variety of early ‘markers’ for autism outcome have been described from 6 months of age (see [22]). We define an early marker as a particular aspect of cognition, behaviour or neural response, measured in infancy, which is associated with later traits of autism. The current study examines the impact of sex on three early markers, which span the domains of social and non-social attention and are known to relate to emerging autistic symptomatology: the Autism Observation Scale for Infants (AOSI; [23, 24]), gaze following behaviour [25] and disengagement latency in the gap-overlap task [26, 27].

The AOSI is an observational assessment of emerging autism-like atypicality measuring, for example, anticipation of social interaction, imitation and motor skills. The second early marker, looking time to the gazed-at object during a gaze following task, is a measure of social attention. Gaze following behaviour can be decomposed into first look (did the infant correctly follow gaze) and looking time (how long did they then spend looking at the object). Looking time is thought to index a more in-depth understanding of the referential meaning of eye-gaze (e.g. [28]) and is the measure which relates to subsequent social-communication abilities [25]. The final marker, disengagement latency from the gap-overlap task, measures infants’ ability to shift attention from a centrally presented stimulus to a peripheral one.

If particular early markers are expressed differently in infant boys and girls, they may act as sex-specific risk or protective factors (Fig. 1a). For example, high-risk girls show greater social attention than high-risk boys—a sex difference in the early marker itself [25]. Alternatively, if no differences are measured in expressed early markers, then sex differences may appear in the relationship between early markers and later traits (Fig. 1b). Differences in the relationship (i.e. the slope) between the marker and autism traits would point to differential liability effects. A significant relationship in boys but not in girls would be consistent with the existence of additional sex-specific factors; these putative factors may confer protection in girls or increase risk in boys.

For all three markers (AOSI, gaze following, disengagement), we first ask whether there are sex differences at 14 months, the age at which these measures have been previously linked with autistic traits in the same cohort of children [25, 26, 23]. More particularly, we ask whether girls show lower AOSI scores, increased attention to the jointly attended object and faster disengagement latencies compared to boys. Second, we test whether the relationship between these infant markers and later autism traits (i.e. Autism Diagnostic Observational Schedule (ADOS) scores) is similar across males and females, predicting a stronger magnitude of relationship between risk and outcome in boys than girls. The ADOS was chosen as the primary outcome because it is both a gold standard measure of autism traits and a continuous outcome measure. This is consistent with the emphasis shift from categorical to more continuous approaches in characterising psychopathology (Diagnostic and Statistical Manual 5th edition (DSM-5) [29]).

## Methods

Ethical approval was given by the National Health Service, National Research Ethics Service London Research Ethics Committee (08/H0718/76), and parents gave informed consent.

### Participants

As part of the British Autism Study of Infant Siblings (BASIS: http://www.basisnetwork.org/), 104 infants (54 high-risk, 21 males; 50 low-risk, 21 males) took part in a battery of assessments at approximately 7 months, 14 months, 2 years and 3 years. At the time of enrolment (<5 months of age), none of the infants had been diagnosed with any medical or developmental condition. Data presented in the current paper come from the 14-month infant visit (mean 13.79 months, SD 1.46; males mean 13.84, SD 1.08; females mean 13.76, SD 1.65) and 3-year outcome visit (mean 37.93 months, SD 3.02; males mean 38.13, SD 3.36; females mean 37.81, SD 2.82). For the high-risk group, consensus ICD-10 [30] *ASD diagnoses* (ASD-sibs; childhood autism; atypical autism, other pervasive developmental disorder (PDD)) were achieved using all available information from all visits by experienced researchers. Seventeen of the high-risk children met ASD criteria (11 males).

Different numbers of infants contributed data to the three early autism markers:

- *Autism Observational Scale for Infants (AOSI)* (see [23]): 53 high-risk (21 males) and 48 low-risk (17 males) infants completed the AOSI assessment (see Table 1).

**Table 1:** Descriptive statistics split by sex and risk group for the 14-month early markers (AOSI, gaze following, disengagement) and 3-year autistic trait measures (ADOS, SCQ)

	AOSI	GF	Disengagement	ADOS	SCQ
	14 months	14 months	14 months	3 years	3 years
	M (SD)	M (SD)	M (SD)	M (SD)	M (SD)
Low risk					
Overall	3.17 (3.25)	0.31 (0.14)	138.15 (105.81)	5.52 (4.33)	3.00 (2.40)
	*N* = 48	*N* = 37	*N* = 46	*N* = 48	*N* = 48
Males	3.59 (3.02)	0.27 (0.08)	148.34 (104.77)	6.41 (5.36)	2.88 (2.12)
	*N* = 17	*N* = 10	*N* = 16	*N* = 17	*N* = 17
Females	2.94 (3.40)	0.32 (0.16)	132.71 (107.73)	5.03 (3.65)	3.06 (2.57)
	*N* = 31	*N* = 27	*N* = 30	*N* = 31	*N* = 31
High risk					
Overall	4.64 (4.47)	0.26 (0.10)	179.55 (152.91)	8.25 (5.34)	6.37 (7.12)
	*N* = 53	*N* = 32	*N* = 52	*N* = 53	*N* = 52
Males	5.19 (5.72)	0.25 (0.12)	196.91 (203.30)	9.24 (5.42)	6.00 (5.33)
	*N* = 21	*N* = 13	*N* = 21	*N* = 21	*N* = 21
Females	4.28 (3.48)	0.26 (0.08)	167.78 (108.81)	7.59 (5.26)	6.61 (8.20)
	*N* = 32	*N* = 19	*N* = 31	*N* = 32	*N* = 31
ANOVA					
Risk group	*F* = 3.26	*F* = 1.86	*F* = 2.36	*F* = 7.19**	*F* = 8.75**
Sex	*F* = 0.92	*F* = 1.11	*F* = 0.77	*F* = 2.26	*F* = 0.12
Risk*sex	*F* = 0.03	*F* = 0.35	*F* = 0.05	*F* = 0.02	*F* = 0.04

*AOSI* Autism Observation Scale for Infants, *GF* gaze following, *disengagement* overlap–baseline saccadic reaction time in gap-overlap task, *ADOS* Autism Diagnostic Observation Schedule total social communication score, *SCQ* Social Communication Questionnaire

**p < 0.01
- *Gaze following task* (see [25]): The same 32 high-risk (13 males) and 37 low-risk (10 males) siblings from the Bedford et al. [25] analysis were included in the current analyses.
- *Gap-overlap task* (see [26]): Data from 52 high-risk siblings (21 males) and 46 low-risk controls (16 males) were included from the gap-overlap task.
- *Autism Diagnostic Observational Schedule-Generic (ADOS-G)*; 3-year ADOS assessments were conducted with 53 high-risk (21 males) and 48 low-risk (17 males) toddlers.
- *Social Communication Questionnaire – Lifetime (SCQ-L)*; Questionnaires were completed for 52 high-risk (21 males) and 48 low-risk (17 males) toddlers.

### Procedure

All the 14-month measures have previously been reported in separate papers—see Gammer et al. [23], Elsabbagh et al. [26] and Bedford et al. [25] for full description of the method.

### Fourteen-month measures

#### Autism Observation Scale for Infants

The Autism Observation Scale for Infants (AOSI; [31]; revised version used in this study [32]) is a semi-structured observational assessment of ASD-related behavioural markers in infancy. A 19-item version of the AOSI was used (see [32]) which gives a total score (sum of all codes; max score 44), with higher scores indexing greater atypicality. The majority of assessments were double coded with excellent reliability (*n* = 85, intraclass correlation coefficient = 0.95).

#### Gaze following task

Infants were seated on their parent’s lap at a distance of 50 cm from the 17-in. flat screen monitor and looking behaviour was recorded using a Tobii 1750 eye-tracker. Stimuli were presented using ClearView software, and gaze data were recorded at 50 Hz. Each trial began with two objects on a table and a female model ‘looking down’ (3 s), then looking up—‘direct gaze’ (2 s)—and then turning her head to look at one of the objects—‘shift’ (6 s). The object looked at by the model during ‘shift’ is the *congruent* object, and the other, non-gazed-at object, is the *incongruent* object. There were 12 trials, and six different pairs of objects counterbalanced across trials.

Trial exclusion criteria were as follows: (1) no looking to the face during ‘direct gaze’ and (2) looking away from the computer screen for the entire ‘shift’ phase. Three high-risk children (none of whom were diagnosed with autism at 3 years) had <3 valid trials and were excluded. *Looking time to the congruent object* was calculated as a proportion (out of total looking time to the slide) during the ‘shift’ phase. A 2*2 ANOVA showed no significant difference in the number of valid trials by group (high- versus low-risk infants: *F*(1, 65) = 1.13, *p* = 0.29), sex (boys versus girls: *F*(1, 65) = 0.001, *p* = 0.98) or group*sex interaction: *F*(1, 65) = 0.09, *p* = 0.77.

#### Gap-overlap task

Infants were seated on their parent’s lap 60 cm from the 46-in. LCD computer screen. A video camera was used to record looking behaviour and trial presentation was controlled by the experimenter. A central stimulus appeared (subtending 13.8° × 18.0°) followed by a peripheral target green balloon (subtending 6.3° × 6.3°) which appeared randomly on the left or right. The target remained on the screen until either (1) the infant looked at it or (2) the maximum time of 2.5 s passed. An animal reward stimulus (elephant, lion, seal, etc.) then appeared in the place of the green balloon. Up to 70 trials were presented depending on infants’ attentiveness. There were two trial types analysed in this study: baseline and overlap. In the baseline condition, the central stimulus disappeared at the *same time* as the peripheral target appeared, whereas in the overlap condition, the central stimulus remained present while the target stimulus appeared in the periphery.

Data were video coded frame-by-frame by two raters (reliability of >0.9 Cohen’s K for trial validity). Invalid trials were those in which the infant (1) looked away from the screen; (2) did not look at the central stimulus immediately before the onset of the peripheral stimulus and (3) looked away or blinked during the onset of the peripheral stimulus. A 2*2 ANOVA showed that there was no significant difference in the number of valid trials by group (high- versus low-risk infants: *F*(1, 94) = 1.77, *p* = 0.19), a marginal effect of sex (boys versus girls: *F*(1, 65) = 3.97, *p* = 0.05) and no group*sex interaction: *F*(1, 65) = 0.06, *p* = 0.81. Because the effect of sex approached significance, with more valid trials for girls, the number of valid trials was included as a covariate in the analyses for disengagement. Saccadic reaction times were analysed from all valid trials in which the infants oriented towards the peripheral stimulus 100–1200 ms after its onset. If the infant did not look towards the stimulus in this time, the trial was called ‘failure to disengage’ and reaction time was not analysed. Disengagement was calculated as reaction time in overlap trials–baseline trials.

### Three-year measures

#### Autism Diagnostic Observational Schedule [33]

The ADOS-G is a semi-structured observational assessment used in the diagnosis of ASD, which involves assessment of social interaction with an examiner through play or conversation (depending on the age of the child), and a range of different behaviours, including language, gestures, eye contact, play and creativity, and stereotyped behaviours are coded. The codes are 0, 1, and 2 (and in some cases 3) with a higher score indicating a greater level of autistic-like atypicality. Certain item scores make up the final ‘algorithm’ scores, which are split into subsections: social, communication, creativity and repetitive and stereotyped behaviours. Ninety-eight children did module 2 and three children completed module 1. At the time the data were collected and first published, the ADOS-G social-communication algorithm was the standard way of computing ADOS scores, and thus we used the social and communication algorithm as a measure of autism traits at 3 years. However, analyses using the new ADOS-2 calibrated social affective severity scores are presented in Additional file 1.

#### Social Communication Questionnaire – Lifetime

The Social Communication Questionnaire (SCQ) is a parent-completed questionnaire with questions developed from the Autism Diagnostic Interview-Revised (ADI-R; [34]).

### Statistical analyses

The infant markers were not significantly correlated with one another in the overall sample (AOSI and gaze following: *r* = −0.17, *p* = 0.17; AOSI and disengagement: *r* = 0.09, *p* = 0.37; gaze following and disengagement: *r* = −0.12, *p* = 0.33). To address our first question—are there sex differences in the early markers, we first ran a 2*2 ANOVA with sex (boys versus girls), group (high-risk versus low-risk) and sex*group interaction for each early marker separately (see Table 1).

To test for sex differences in the relationship between early markers and later ADOS outcome, we first explored lowess curves (smoothed lines through the points on the scatterplot) to check that there was overlap in the data between boys and girls. Based on visual inspection of the plots, any extreme non-overlapping values were trimmed to one point above the next highest score [35]. The relationship between the predictor and outcome can differ along the predictor’s scale (i.e. a non-linear relationship). Restricting the analysis to the region where the predictors overlap in boys and girls ensures that any sex differences in the relationships between early markers and later autism traits are not due to the groups spanning different portions of the predictor scale (see [36] for discussion of this issue). Analyses followed the same procedure for each early marker: (1) linear regression with ADOS social and communication algorithm score as the outcome and early marker, and sex and early marker*sex interaction as predictors; (2) planned sex comparisons: linear regression of ADOS score on predictor split by sex.

## Results

### Autism Observation Scale for Infants total score

A 2*2 ANOVA showed no significant main effect of sex on total AOSI score at 14 months: *F*(1, 97) = 0.92, *p* = 0.34, *ηp*^2^ = 0.01 (see Table 1). The risk group effect was marginally significant *F*(1, 97) = 3.26, *p* = 0.07, *ηp*^2^ = 0.03, and there was no group*sex interaction *F*(1, 97) = 0.03, *p* = 0.88, *ηp*^2^ < 0.001.

**Fig. 2:**
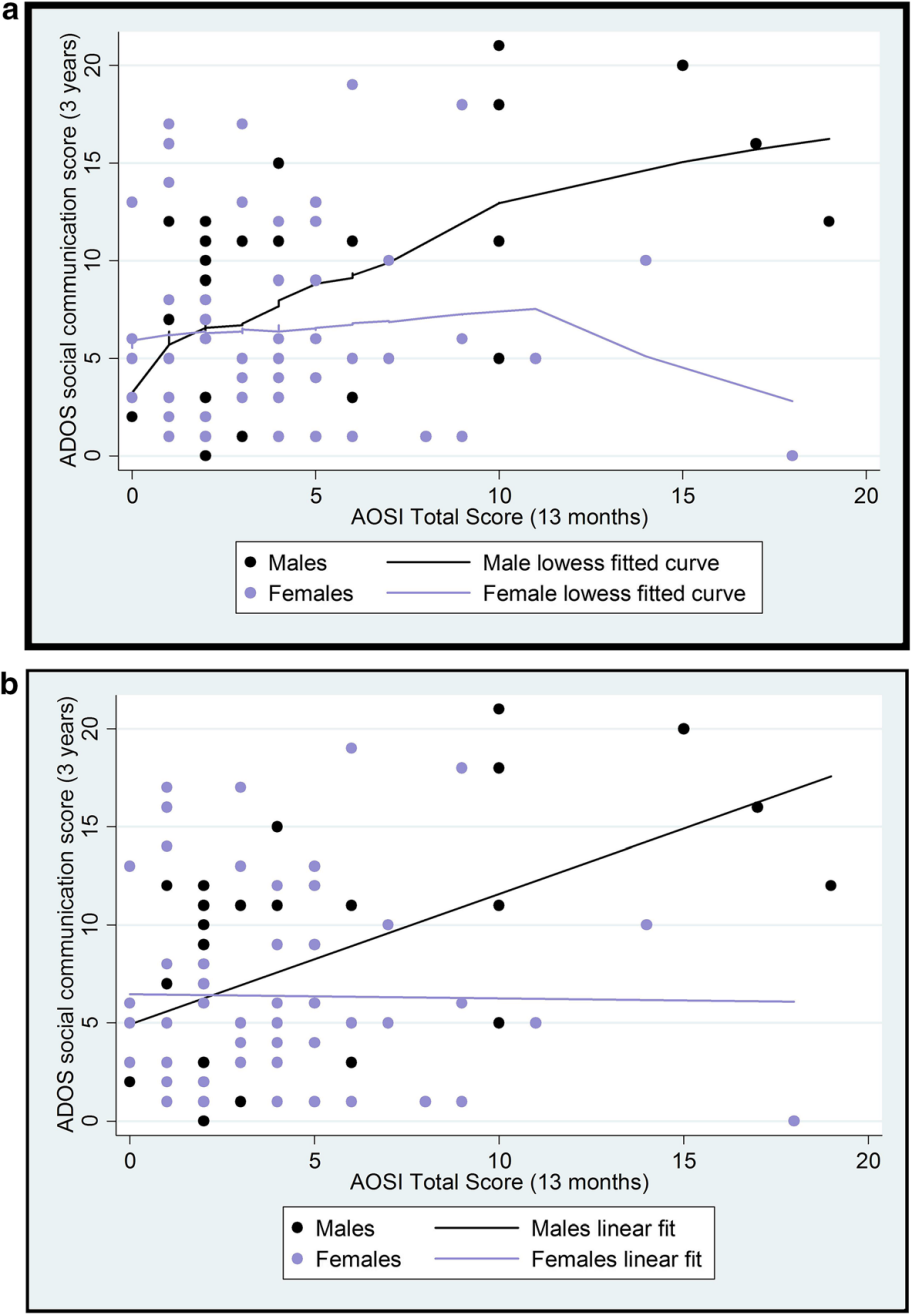
**a** The relationship between infant AOSI and 3-year ADOS outcome with smoothed lowess curves for males and females. **b** The relationship between infant AOSI and 3-year ADOS outcome with linear fit for males and females

A smoothed lowess curve indicated good overlap between males and females across the range of scores (see Fig. 2). A linear regression showed a significant relationship between AOSI and ADOS score (*β* = 0.527, *p* < 0.001) and a significant sex*AOSI interaction (*β* = −0.44, *p* = 0.005). When this was broken down by sex, AOSI was a significant predictor of ADOS in males (*β* = 0.57, *p* < 0.001) but not in females (*β* = −0.015, *p* = 0.905). Results were similar across high- and low-risk groups with significant effects in males (high risk: *β* = 0.588, *p* = 0.005; low risk: *β* = 0.542, *p* = 0.03) but not in females (high risk: *β* = −0.29, *p* = 0.113; low risk: *β* = 0.059, *p* = 0.75). Significance levels remained unchanged when Mullen Scales of Early Learning (MSEL) verbal and non-verbal *t* scores were added as covariates (see Additional file 1).

### Gaze following

Results from a 2*2 ANOVA showed that there was no significant effect of sex on correct looking time from a gaze following task: *F*(1, 65) = 1.11, *p* = 0.30, *ηp*^2^ = 0.02 (see Table 1). The risk group effect (*F*(1, 65) = 1.86, *p* = 0.18, *ηp*^2^ = 0.03) and group*sex interaction (*F*(1, 65) = 0.35, *p* = 0.56, *ηp*^2^ = 0.005) were also not significant.

**Fig. 3:**
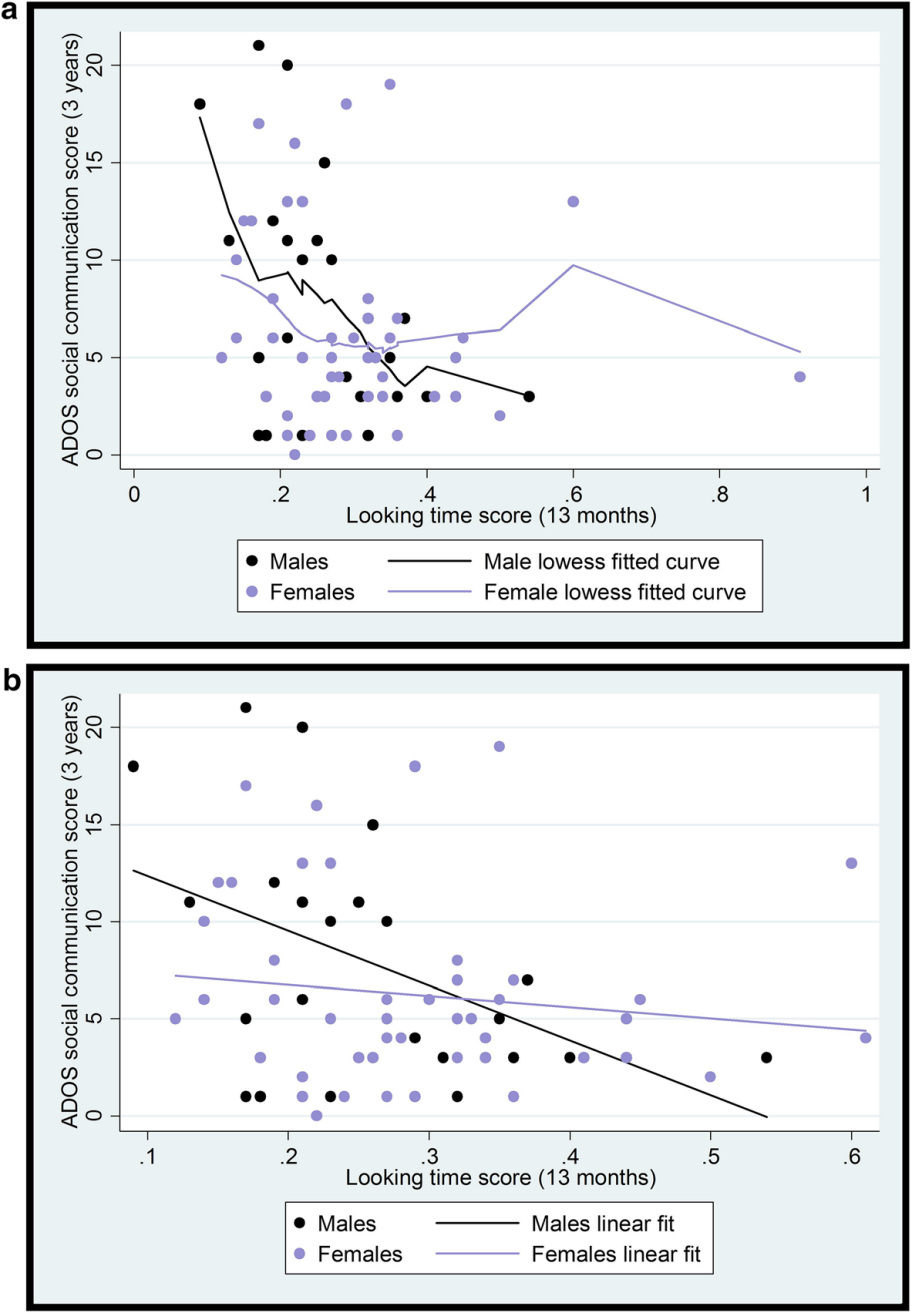
**a** The relationship between infant looking time in the gaze following task and 3-year ADOS outcome with smoothed lowess curves for males and females. For further analysis, the outlier was trimmed back to one point above the next highest value. **b** The relationship between infant looking time in the gaze following task and 3-year ADOS outcome with linear fit for males and females

Examination of the lowess curves (see Fig. 3a) showed that at high levels of gaze time there were no data for males due to an outlier, a female with particularly long looking time. For the following analysis, this point was trimmed back to 0.61 (the value one point above the highest non-outlier score, see [35]).

Gaze time significantly predicted ADOS score (*β* = −0.567, *p* = 0.012), while the effect of sex (*β* = −0.64, *p* = 0.053) and the sex*gaze time interaction time (*β* = 0.689, *p* = 0.086) were marginally significant. When we ran separate simple linear regressions for males and females, gaze time predicted later ADOS in males (*β* = −0.46; *F*(1,21) = 5.64, *p* = 0.027; *R*^2^ = 0.21) but not in females (*β* = −0.13, *p* = 0.39). We did not look at this analysis split by risk group owing to the very small sample size for the males (*n* = 10 low risk, *n* = 13 high risk). Results remained substantively similar when MSEL scores were added as covariates (see Additional file 1).

### Disengagement

For disengagement, number of valid trials was included as a covariate in the analysis, to account for the marginally significant sex effect. As for the other infant markers, no significant sex difference in disengagement was found *F*(1, 93) = 0.77, *p* = 0.38, *ηp*^2^ = 0.008 in an ANOVA. The main effect of risk group *F*(1, 93) = 2.36, *p* = 0.13, *ηp*^2^ = 0.03 and the group*sex interaction were also non-significant *F*(1, 93) = 0.05, *p* = 0.82, *ηp*^2^ = 0.001. There was no effect of the number of valid trials (*F*(1, 93) = 0.23, *p* = 0.64, *ηp*^2^ = 0.002).

**Fig. 4:**
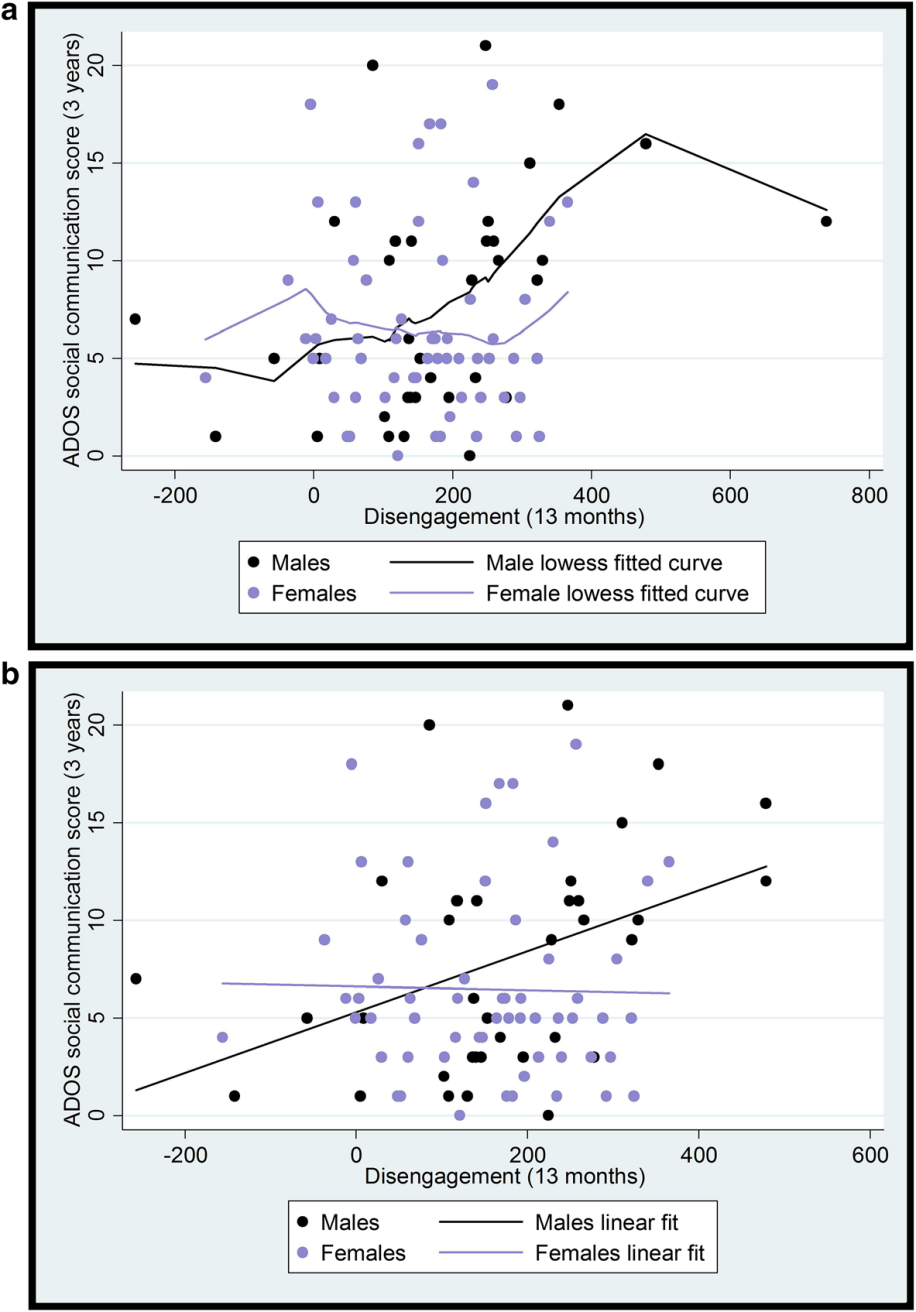
**a** The relationship between infant disengagement in the gap-overlap task and 3-year ADOS outcome with smoothed lowess curves for males and females. For further analysis, the outlier was trimmed back to one point above the next highest value. **b** The relationship between infant disengagement in the gap-overlap task and 3-year ADOS outcome with linear fit for males and females

Similarly to the gaze time measure, examination of the lowess curve suggested a region of no overlap between male and female disengagement scores at very slow RTs, owing to a single unmatched point. For the following analysis, this point was trimmed to a value of 479 ms (see Fig. 4).

When entered into a regression model, disengagement reaction time was a significant predictor of subsequent ADOS score (*β* = 0.38, *p* = 0.007). There was no effect of valid disengagement trials (*β* = −0.05, *p* = 0.66). The interaction between sex and disengagement was marginally significant (*β* = −0.36, *p* = 0.05), and when this was broken down into two separate regressions, again the relationship between disengagement and ADOS score was significant for males (*β* = 0.41, *p* = 0.013) but not for females (*β* = −0.02, *p* = 0.86). When split by risk group, the *β* values remained very similar with the effect of disengagement on ADOS in males becoming marginally significant in the high-risk group (*β* = 0.43, *p* = 0.06) and non-significant for the low-risk group (*β* = 0.39, *p* = 0.18). Neither group showed a significant effect in females (*p* values >0.66). Again, results remained similar after controlling for MSEL scores (see Additional file 1).

### Social Communication Questionnaire analysis

To confirm that our results were not due to some specific measurement issue related to the ADOS, we also assessed the relationship between risk factors and parent-reported SCQ score. As the data were skewed, a square root transformation was also applied to the SCQ and both transformed and untransformed results are presented. Results remained similar, with the AOSI scores significantly predicting SCQ scores in males (*β* = 0.35, *p* = 0.04; although this became a trend only for the transformed scores *β* = 0.26, *p* = 0.12) but not in females (*β* = 0.07, *p* = 0.61; transformed *β* = 0.08, *p* = 0.53). Gaze following behaviour was marginally significant in males (*β* = −0.38, *p* = 0.08; transformed *β* = −0.37, *p* = 0.08) and not significant in females (*β* = 0.08, *p* = 0.59; transformed *β* = 0.12, *p* = 0.44), and disengagement latency significantly predicted SCQ in males (*β* = 0.35, *p* = 0.04; transformed *β* = 0.32, *p* = 0.06) but not in females (*β* = 0.14, *p* = 0.32 transformed *β* = 0.13, *p* = 0.33).

## Discussion

The aim of this paper was to investigate sex differences in three documented early autism markers, which are known to associate with the severity of autistic social-communication symptomatology, at 3 years of age. We found no evidence for sex differences in the AOSI severity scores, gaze following and attentional disengagement, when infants were 14 months of age. However, these measures appeared to act as early markers *in boys only*, with all three significantly relating to later 3-year ADOS social communication and SCQ scores in males but not in females.

Our results show that so-called early autism markers, previously shown to be associated with later autistic symptomatology [25, 26, 23], are actually only effective markers in the boys. However, since there are no sex differences in the expression of these behaviours at 14 months, this suggests the existence of additional moderating factors. One line of evidence which could help to determine whether boys have additional risk factors, or girls additional protective factors, comes from looking at the risk group effects. If the there is a main effect of risk group (where high-risk boys *and* girls show increased early markers compared to low-risk boys and girls), this would suggest that perhaps the females recover from risk, i.e. they benefit from protective factors. Alternatively, if the groups show similar levels of these early markers, this suggests that the boys have additional risk factors. Our results show no significant risk group effects for the three markers, although, as there are trend effects for each measure, this cannot be used as strong evidence to tease apart increased risk versus protection. What we can conclude is that there must be additional sex-specific factors which moderate the developmental effects of the early markers.

Genetic susceptibility effects have often been described in psychopathology, where particular gene variants confer more or less susceptibility to the effects of other genes (e.g. [37]) or particular environments [38]. Given the variety of the early markers we examined, which span the social and non-social domains of the early autism phenotype, any moderating factors that might increase the impact of these traits on emerging pathology in boys (or that decrease their impact in girls) will need to have a broad scope. Messinger et al. [5] found sex differences in Mullen Scales for Early Learning (MSEL) scores in both low-risk and high-risk participants. From 18 months (the earliest age included in analysis), girls outperformed boys, across the subscales (expressive and receptive language, visual reception, fine motor). Faster general development could protect girls against the effect of other risk factors. However, despite replicating these sex differences in our cohort, covarying for MSEL scores did not alter the differential relationship between early markers and outcomes. Other potential moderating factors will have to be explored in the future. For example, one recent hypothesis proposes that sex differences in synaptic and regional plasticity could explain differences in autism prevalence rates [39].

It is also possible that interactions occur between the markers under investigation, i.e. that a combination of higher AOSI scores, reduced gaze following and slower disengagement is necessary for girls to manifest autism symptoms. Indeed, it was shown that gaze following and attention disengagement act additively in predicting later autism outcome (e.g. [40]). It could be that girls need multiple hits across several different factors, consistent with the existence of additional protective factors in girls, whereas boys are more susceptible to a single-hit pathway [41]. Future studies with larger sample sizes will be important to test for interactive developmental effects.

Given that we found no early sex differences in early markers at 14 months, i.e. quite late in development, one interesting possibility is that some sex-specific moderating factors may be environmental. In typical infants, differences in maternal social interaction with boys and girls have been documented, with different styles of sensitive responding (e.g. [42]), and increased parent-infant communication in response to infant girls than boys, even in the absence of behavioural differences between the infants themselves [43, 44]. Parent-child interaction is known to be important in the development of socio-communication abilities, including in autism [45], although it remains unknown whether the above sex differences in parental interaction are consequential for development. However, protracted expression of intrinsic moderating factors is also possible. For example, while sex differences in neural gene expression peak prenatally, the effects of certain genes continue on after birth into early childhood [46].

Our results were similar across high-risk and low-risk groups indicating that the link with later social-communication abilities is not specific to those at risk for autism. This suggests that our markers reflect common variation as proposed by recent genetic models of autism [47]. The results are consistent with Messinger et al.’s [5] study, who found that sex differences in toddlers with autism reflect typically occurring sex differences also seen in low-risk controls. Other neurodevelopmental disorders such as ADHD and early onset antisocial behaviour disorder also show an increased prevalence in males [2], and future research will be required to study sex differences in the broader context of psychopathology and development, since it is unlikely that sex-specific mechanisms are disorder-specific. There is evidence, for example, of sex differences in the relationship between sensitive parenting during infancy and the subsequent development of callous unemotional (CU) traits (e.g. [48, 49]) with increased maternal sensitivity associated with lower CU traits most strongly in girls.

A clear strength of the current paper is the use of a prospective infant sibling design. This allows us to test for sex differences without the male ascertainment bias faced by studies with diagnosed individuals [5]. However, given our modest sample size, there is a clear need for replication. While we specifically use two clinical tools, the ADOS and SCQ, to deal with tool-specific lack of sensitivity, it remains possible that neither of these instruments capture autistic traits in girls at 3 years of age, when they were administered. Future work assessing symptoms of autism measured later in childhood should establish whether these sex-specific relationships are time dependent. In addition, a larger sample would enable the relationships with categorical autism diagnostic outcomes to be assessed.

The results of the current study draw attention to the need to routinely examine sex differences in the prediction of later autistic symptoms. It is plausible that factors which have previously been found to be non-significant in the prediction of autism symptoms may in fact be associated in the boys, but the overall effect is masked by the presence of girls. However, although we find that three different measures previously described as early markers for autism are only really markers of later autistic traits in boys, this should not encourage research to selectively study this sex. While investigating only boys will increase prediction [50], studying girls is crucial not only for tailoring diagnosis and intervention to this smaller but by no means negligible group but also for unveiling potential important protection that could be used to improve outcomes for both boys and girls.

## Conclusions

In conclusion, although we found no evidence for early sex differences in markers previously shown to relate to emerging autistic symptoms, we did find sex-specific relationships with later autistic traits, with prediction to outcome in males but not in females. These data suggest that so-called early autism markers may only act as markers in boys. This implies the existence of additional sex-specific intervening factors, yet to be identified, which confer risk in boys or protection in girls. Our results emphasise the importance of testing for a moderating effect of sex on emerging autism traits. Characterising sex differences in the developmental trajectory leading to autism has important potential implications for differential early identification of autism in boys and girls.

## Additional file

Additional file 1: Supplementary information. (DOCX 31 kb)

## Abbreviations

ADI-R: Autism Diagnostic Interview-Revised
ADOS: Autism Diagnostic Observational Schedule
AOSI: Autism Observation Scale for Infants
ASD: autism spectrum disorder
BASIS: British Autism Study of Infant Siblings
DSM-5: Diagnostic and Statistical Manual 5th edition
MSEL: Mullen Scales of Early Learning
SCQ: Social Communication Questionnaire
