# Erratum to: Sex differences in the association between infant markers and later autistic traits

**DOI:** 10.1186/s13229-016-0094-8

**Published:** 2016-06-30

**Authors:** Rachael Bedford, Emily J. H. Jones, Mark H. Johnson, Andrew Pickles, Tony Charman, Teodora Gliga

**Affiliations:** Biostatistics Department, Institute of Psychiatry, Psychology & Neuroscience, King’s College London, London, UK; Centre for Brain and Cognitive Development, Birkbeck College, University of London, London, UK; Psychology Department, Institute of Psychiatry, Psychology & Neuroscience, King’s College London, London, UK

## Erratum

After publication of the article [1], the authors noted that an error was observed in the coding of one variable in the analysis. Three children from the low-risk control group were coded as girls in the published version but were actually boys. After re-running all the statistical analyses, the effects we reported remain substantially similar, and therefore the conclusions of the manuscript and our summary in the abstract remain unchanged. Detailed corrected analyses are presented below.

### Participants

Data presented in the current paper come from the 14 month infant visit (mean 13.79 months, SD 1.46; males mean 13.73, SD 1.16; females mean 13.83, SD 1.64) and 3 year outcome visit (mean 37.93 months, SD 3.02; males mean 37.98, SD 3.29, females mean 37.90, SD 2.85). For the high-risk group, consensus ICD-10 (World Health Organization, 1993) *ASD diagnoses* (ASD-sibs; childhood autism; atypical autism, other pervasive developmental disorder, PDD) were achieved using all available information from all visits by experienced researchers. Seventeen of the high-risk children met ASD criteria (11 male).

Different numbers of infants contributed data to the three early autism markers, as such:

*Autism Observational Scale for Infants (AOSI);* (see Gammer et al., 2015): 53 high-risk (21 male) and 48 low-risk (20 male) infants completed the AOSI assessment (see Table 1).

**Table 1:** Descriptive statistics split by sex and risk group for the 14 month early markers (AOSI, Gaze Following, Disengagement) and 3 year autistic trait measures (ADOS, SCQ)

	AOSI 14 months M (SD)	GF 14 months M (SD)	Disengagement 14 months M (SD)	ADOS 3 years M (SD)	SCQ 3 years M (SD)
Low risk Overall	3.17 (3.25)	0.31 (0.14)	138.15 (105.81)	5.52 (4.33)	3.00 (2.40)
	*N* = 48	*N* = 37	*N* = 46	*N* = 48	*N* = 48
Males	3.30 (2.94)	0.27 (0.09)	153.74 (103.78)	5.90 (5.12)	3.00 (1.97)
	*N* = 20	*N* = 13	*N* = 19	*N* = 20	*N* = 20
Females	3.07 (3.51)	0.33 (0.16)	127.17 (107.78)	5.25 (3.74)	3.00 (2.69)
	*N* = 28	*N* = 24	*N* = 27	*N* = 28	*N* = 28
High risk Overall	4.64 (4.47)	0.26 (0.10)	179.55 (152.91)	8.25 (5.34)	6.37 (7.12)
	*N* = 53	*N* = 32	*N* = 52	*N* = 53	*N* = 52
Males	5.19 (5.72)	0.25 (0.12)	196.91 (203.30)	9.24 (5.42)	6.00 (5.33)
	*N* = 21	*N* = 13	*N* = 21	*N* = 21	*N* = 21
Females	4.28 (3.48)	0.26 (0.08)	167.78 (108.81)	7.59 (5.26)	6.61 (8.20)
	*N* = 32	*N* = 19	*N* = 31	*N* = 32	*N* = 31
ANOVA					
Risk group	*F* = 3.71	*F* = 2.39	*F* = 2.32	*F* = 8.20**	*F* = 8.89**
Sex	*F* = 0.50	*F* = 1.28	*F* = 1.03	*F* = 1.34	*F* = 0.08
Risk*sex	*F* = 0.18	*F* = 0.43	*F* = 0.002	*F* = 0.25	*F* = 0.08

AOSI – Autism Observation Scale for Infants; GF – Gaze Following; Disengagement – Overlap – Baseline saccadic reaction time in Gap-overlap task; ADOS – Autism Diagnostic Observation Schedule total social communication score; SCQ – Social Communication Questionnaire. **p < 0.01

*Gaze following task;* (see Bedford et al., 2012): The same 32 high-risk siblings (13 male) and 37 low-risk (13 male) from the Bedford et al. (2012) analysis were included in the current analyses. *Gap-overlap task;* (see Elsabbagh et al., 2013): Data from 52 high-risk siblings (21 male) and 46 low-risk controls (19 male) were included from the gap-overlap task. *Autism Diagnostic Observational Schedule (ADOS-G)*; Three-year ADOS assessments were conducted with 53 high-risk toddlers (21 male) and 48 low risk (20 male). *Social Communication Questionnaire – Lifetime (SCQ-L);* Questionnaires were completed for 52 high-risk toddlers (21 male) and 48 low risk (20 male).

### Fourteen month measures, data reduction

#### Gaze following task

A 2*2 ANOVA showed no significant difference in the number of valid trials by group (high- versus low- risk infants: F(1, 65) = 1.40, p = 0.24), sex (boys versus girls: F(1, 65) = 0.10, p = 0.76) or group*sex interaction: F(1, 65) = 0.41, p = 0.52.

#### Gap-overlap task

A 2*2 ANOVA showed that there was no significant difference in the number of valid trials by group (high- versus low- risk infants: F(1, 94) = 2.46, *p* = 0.12), sex (boys versus girls: F(1, 94) = 2.43, *p* = 0.12) and no group*sex interaction: F(1, 94) = 0.04, *p* = 0.84.

## Results

### AOSI total score

A 2*2 ANOVA showed no significant main effect of sex on total AOSI score at 14 months: F(1, 97) = 0.5, *p* = 0.48, ηp^2^ = 0.005 (see Table 1). The risk group effect was marginally significant F(1, 97) = 3.71, *p* = 0.06, ηp^2^ = 0.04, and there was no group*sex interaction F(1, 97) = 0.18, *p* = 0.67, ηp^2^ = 0.002.

A linear regression showed a significant relationship between AOSI and ADOS score (β = 0.54, *p* < 0.001) and a significant sex*AOSI interaction (β = -0.47, *p* = 0.003). When this was broken down by sex, AOSI was a significant predictor of ADOS in males (β = 0.58, *p* < 0.001) but not females (β = -0.03, *p* = 0.81) see Figure 2. Results were similar across high and low risk groups with significant effects in males (high risk: β = 0.588, *p* = 0.005; low risk: β = 0.53, *p* = 0.02) but not females (high risk: β = 0.059, p = 0.75; low risk: β = -0.31, *p* = 0.11). Significance levels remained unchanged when Mullen Scales of Early Learning (MSEL) verbal and non-verbal t-scores were added as covariates (see supplementary information).

**Fig. 2:**
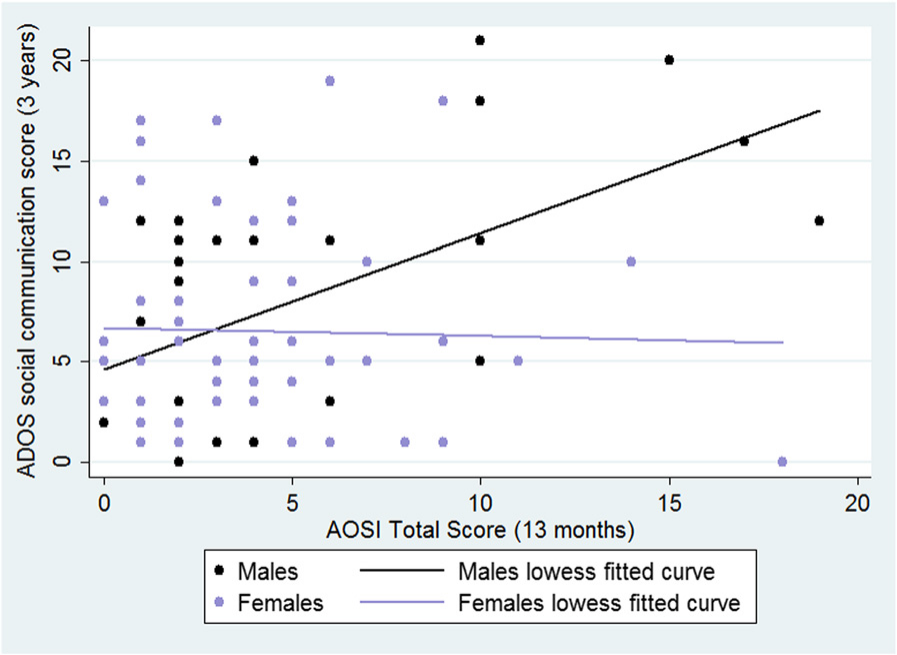
The relationship between infant AOSI and 3 year ADOS outcome with linear fit for males and females

### Gaze following

Results from a 2*2 ANOVA showed that there was no significant effect of sex on correct looking time from a gaze following task: F(1, 65) = 1.28, *p* = 0.26, ηp^2^ = 0.02 (see Table 1). The risk group effect (F(1, 65) = 2.39, *p* = 0.13, ηp^2^ = 0.04) and group*sex interaction (F(1, 65) = 0.43, *p* = 0.52, ηp^2^ = 0.006) were also not significant.

Gaze time significantly predicted ADOS score (β = -0.51, *p* = 0.01), while the effect of sex (β = -0.52, *p* = 0.11) and the sex*gaze time interaction time (β = 0.60, *p* = 0.12) did not reach significance. When we ran separate simple linear regressions for males and females, gaze time predicted later ADOS in males (β = -0.44, *p* = 0.024) but not females (β = -0.13, *p* = 0.41) see Figure 3. We did not look at this analysis split by risk group owing to the very small sample size for the males (*n* = 13 low risk, *n* = 13 high risk). Results remained substantively similar when MSEL scores were added as covariates (see supplementary information).

**Fig. 3:**
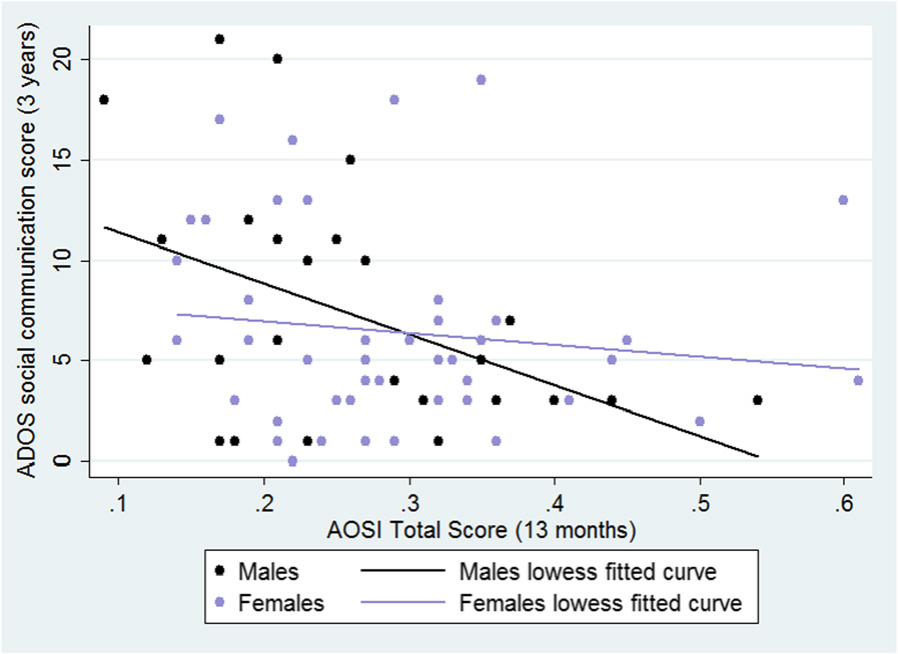
The relationship between infant Looking Time in the Gaze Following Task and 3-year ADOS outcome with linear fit for males and females

### Disengagement

For disengagement, as for the other infant markers, no significant sex difference in disengagement was found F(1, 94) = 1.03, *p* = 0.31, ηp^2^ = 0.01. The main effect of risk group F(1, 94) = 2.32, *p* = 0.13, ηp^2^ = 0.02 and the group*sex interaction were also non-significant F(1, 94) = 0.002, *p* = 0.96, ηp^2^ < 0.001.

When entered into a regression model, disengagement reaction time was a significant predictor of subsequent ADOS score (β = 0.36, *p* = 0.01). The interaction between sex and disengagement was marginally significant (β = -0.32, *p* = 0.08) and when this was broken down into two separate regressions, again the relationship between disengagement and ADOS score was significant for males (β = 0.38, *p* = 0.017) but not females (β = -0.003, *p* = 0.98) see Figure 4. When split by risk group the β values remained very similar with the effect of disengagement on ADOS in males becoming marginally significant in the high risk group (β = 0.41, *p* = 0.07) and non-significant for the low risk group (β = 0.31, *p* = 0.22). Neither group showed a significant effect in females (*p* values > 0.73). Again, results remained similar after controlling for MSEL scores (see supplementary information).

**Fig. 4:**
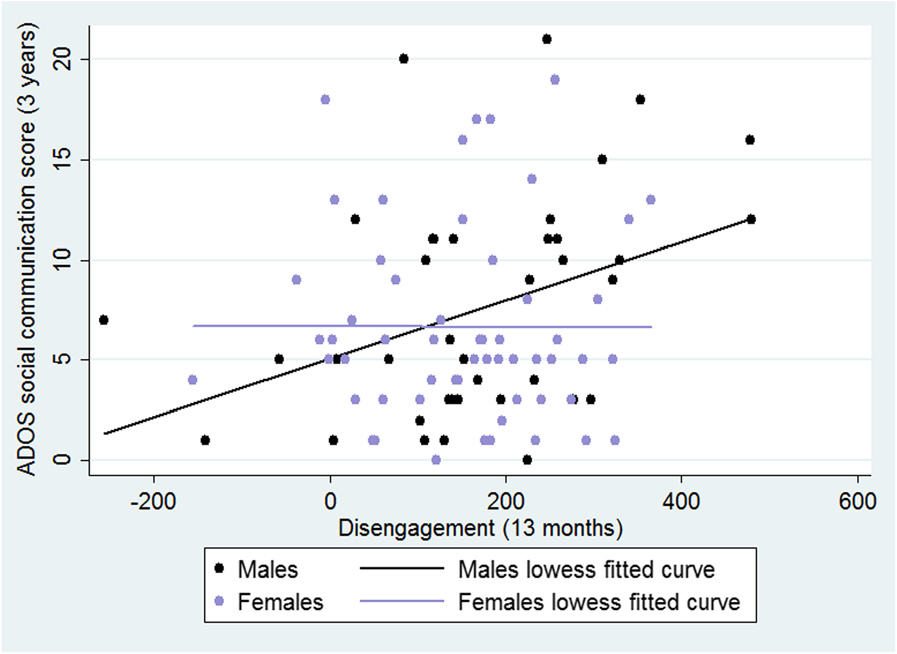
The relationship between infant disengagement in the gap-overlap task and 3 year ADOS outcome with linear fit for males and females

### Social Communication Questionnaire (SCQ) analysis

To confirm that our results were not due to some specific measurement issue related to the ADOS, we also assessed the relationship between risk factors and parent reported SCQ score. As the data were skewed, a square root transformation was also applied to the SCQ and both transformed and untransformed results are presented. Results remained similar, with the AOSI scores significantly predicting SCQ scores in males (β = 0.35, *p* = 0.03; although this became a trend only for the transformed scores β = 0.25, *p* = 0.12) but not females (β = 0.06, *p* = 0.63; transformed β = 0.09, *p* = 0.52). Gaze following behaviour was marginally significant in males (β = -0.34, *p* = 0.09; transformed β = -0.33, *p* = 0.10) and not significant for females (β = 0.09, *p* = 0.59; transformed β = 0.12, *p* = 0.43), and disengagement latency significantly predicted SCQ in males (β = 0.34, p = 0.03; transformed β = 0.31, *p* = 0.05) but not females (β = 0.14, *p* = 0.30; transformed β = 0.14, *p* = 0.32).
