# Supplementary material for: Sex differences in the association between infant markers and later autistic traits
Source: Mol Autism. 2016 Mar 30;7:21. doi: 10.1186/s13229-016-0081-0 (PMC4815081; doi:10.1186/s13229-016-0081-0)
Supplement: Additional file 1: — Supplementary information. (DOCX 31 kb) [file 13229_2016_81_MOESM1_ESM.docx]

Supplementary Information

*Mullen Scales of Early Learning (MSEL)*

The Mullen Scales of Early Learning (MSEL) is a standardised developmental assessment, which characterises early motor and cognitive development from 0-68 months. The assessment is comprised of five subscales: gross motor (GM), visual reception (VR), fine motor (FM), receptive language (RL) and expressive language (EL). We computed verbal (receptive + expressive T-scores /2) and non-verbal (visual reception + fine motor T-scores/2) scores.

As there were main effects of risk group and sex for MSEL scores (see Table S1), replicating Messinger et al.’s (2015) study, we re-ran the analysis to include MSEL verbal and non-verbal scores as covariates in the regression analysis. Results remained similar, with the sex*early marker interaction remaining significant for the AOSI (β = -0.433, p = 0.008), and marginally significant for disengagement (β = -0.34, p = 0.07) and gaze following (β = 0.65, p = 0.11). Linear regressions split by sex, with the early marker and MSEL scores as predictors and ADOS score as the outcome, showed significant relationships for AOSI, gaze following and disengagement only in the boys (p values < 0.05), with no significant relationships in the girls (p values > 0.47).

*ADOS Calibrated Social Affective Severity Scores*

Because 3/98 children completed module 1, we re-ran the analysis using ADOS calibrated social affective severity scores as the outcome, which allow more easily for direct comparison across modules. Again, the results remain substantively similar, with a significant sex*early marker interaction for AOSI (β = 0.45, p = 0.02), disengagement (β = -0.39, p = 0.04) and a marginal effect for gaze following (β = 0.70, p = 0.09). Posthoc tests show significant effects for all predictors in boys (p values < 0.023) but not in girls (p values > 0.49).

*Table S1* Correlation matrix showing relationships between early markers and later autism traits

|  | AOSI | Disengagement | Gaze Time | ADOS |
| --- | --- | --- | --- | --- |
| AOSI | - |  |  |  |
| Disengagement | r = 0.09  p = 0.37 | - |  |  |
| Gaze Time | r = -0.17  p = 0.17 | r = -0.12  p = 0.33 | - |  |
| ADOS | r = 0.28  p = 0.005 | r = 0.21  p = 0.05 | r = -0.24  p = 0.04 | - |
| SCQ | r = 0.16  p = 0.12 | r = 0.18  p = 0.07 | r = 0.01  p = 0.93 | r = 0.08  p = 0.45 |

# *Table S2* Descriptive statistics split by sex and risk group for the 14 month and 3 year Mullen Scales of Early Learning verbal and non-verbal T-scores.

|  | MSEL verbal  14 months  M (SD) | MSEL non-verbal  14 months  M (SD) | MSEL verbal  3 years  M (SD) | MSEL non-verbal  3 years  M (SD) |
| --- | --- | --- | --- | --- |
| Low risk  Overall  Males  Females | 47.09 (9.56)  N = 48  42.29 (10.38)  N = 17  49.73 (8.09)  N = 31 | 58.44 (8.26)  N = 47  56.16 (9.38)  N = 16  59.61 (7.51)  N = 31 | 58.19 (8.72)  N = 48  54.53 (8.68)  N = 17  60.19 (8.20)  N = 31 | 57.79 (9.90)  N = 48  53.06 (10.82)  N = 17  60.39 (8.45)  N = 31 |
| High risk  Overall  Males  Females | 43.89 (10.96)  N = 53  37.93 (8.90)  N = 21  47.80 (10.52)  N = 32 | 53.07 (10.30)  N = 53  49.40 (8.15)  N = 21  55.47 (10.96)  N = 32 | 51.92 (11.79)  N = 53  47.83 (13.19)  N = 21  54.61 (10.12)  N = 32 | 52.56 (13.02)  N = 52  47.35 (13.51)  N = 20  55.81 (11.76)  N = 32 |
| **ANOVA****Risk group****Sex** Risk*sex | F=2.6F=19.6** F=0.39 | F=8.14**F=6.22* F=0.47 | F=8.72**F=8.95** F=0.07 | F=5.00*F=11.79** F=0.06 |

# * p<0.05, ** p<0.01

MSEL – Mullen Scales of Early Learning

*Figure S1* Histogram of ADOS Social & Communication algorithm scores split by sex.

*Figure S2* Histograms of SCQ total and square root transformed scores split by sex.
